# Sex‐based differences in antiretroviral therapy initiation, switching and treatment interruptions: global overview from the International Epidemiologic Databases to Evaluate AIDS (IeDEA)

**DOI:** 10.1002/jia2.25149

**Published:** 2018-06-29

**Authors:** Michelle L Giles, Amit C Achhra, Alison G Abraham, Andreas D Haas, Michael John Gill, Man Po Lee, Marco Luque, Catherine McGowan, Morna Cornell, Paula Braitstein, Nathalie de Rekeneire, Renaud Becquet, Kara Wools‐Kaloustian, Matthew Law

**Affiliations:** ^1^ Department of Infectious Diseases Monash University and Alfred Health Melbourne VIC Australia; ^2^ Kirby Institute UNSW Sydney Australia; ^3^ John Hopkins School of Medicine Baltimore MA USA; ^4^ University of Bern Bern Switzerland; ^5^ University of Calgary Calgary AB Canada; ^6^ Queen Elizabeth Hospital Hong Kong China; ^7^ Hospital Escuela Universitario Tegucigalpa Honduras; ^8^ Vanderbilt University Nashville TN USA; ^9^ Centre for Infectious Disease Epidemiology and Division of Epidemiology and Biostatistics School of Public Health and Family Medicine University of Cape Town Cape Town South Africa; ^10^ University of Toronto Toronto ON Canada; ^11^ University of Bordeaux Inserm Bordeaux Population Health Research Center UMR 1219 Team IDLIC Bordeaux France; ^12^ Indiana University School of Medicine Indianapolis IN USA

**Keywords:** cohort studies, gender, treatment, women, sex, HIV

## Abstract

**Introduction:**

In 2015, the World Health Organization recommended that all HIV‐infected individuals consider ART initiation as soon as possible after diagnosis. Sex differences in choice of initial ART regimen, indications for switching, time to switching and choice of second‐line regimens have not been well described. The aims of this study were to describe first‐line ART and CD4 count at ART initiation by sex, calendar year and region, and to analyse time to change or interruption in first‐line ART, according to sex in each region.

**Methods:**

Participating cohorts included: Southern, East and West Africa (IeDEA‐Africa), North America (NA‐ACCORD), Caribbean, Central/South America (CCASAnet) and Asia‐Pacific including Australia (IeDEA Asia‐Pacific). The primary outcomes analysed for each region and according to sex were choice of initial ART, time to switching and time to discontinuation of the first‐line regimen.

**Results and Discussion:**

The combined cohort data set comprised of 715,252 participants across seven regions from low‐ to high‐income settings. The median CD4 count at treatment initiation was lower in men compared with women in nearly all regions and time periods. Women from North America and Southern Africa were more likely to switch ART compared to men (*p* < 0.001) with approximately 90% of women reporting a major change after 10 years in North America. Overall, after 8 years on ART, >50% of HIV‐ positive men and women from Southern Africa, East Africa, South and Central America remained on their original regimen. Men were more likely to have a treatment interruption compared with women in low‐ and middle‐income countries from the Asia/Pacific region (*p* < 0.001) as were men from Southern Africa (*p* < 0.001). Greater than 75% of men and women did not report a treatment interruption after 10 years on ART from all regions except North America and Southern Africa.

**Conclusions:**

There are regional variations in the ART regimen commenced at baseline and rates of major change and treatment interruption according to sex. Some of this is likely to reflect changes in local and international antiretroviral guideline recommendations but other sex‐specific factors such as pregnancy may contribute to these differences.

## 1 Introduction

Over the past decade, HIV treatment guidelines have changed, with the CD4^+^ count threshold for initiating antiretroviral therapy (ART) gradually increasing from less than 350 cells/μL in 2010 to <500 cells/μL in 2013 1. In 2014, UNAIDS launched the “90‐90‐90” goals to increase to 90% by 2020 the proportion of persons living with HIV infection who know their status, the proportion of persons living with HIV infection receiving ART and the proportion of persons living with HIV infection on ART who have achieved viral suppression 2. More recently, in September 2015, the World Health Organization (WHO) announced that all persons infected with HIV should begin ART as soon as possible after diagnosis, thereby removing eligibility criteria for all populations and age groups (http://www.who.int).

Accompanying these recommendations, expanded ART programmes and wider availability of virological monitoring 3 are making quality care more accessible globally, including lower income countries. It can be anticipated that earlier treatment initiation and wider access to ART will decrease HIV‐related mortality further and result in an ever increasing number of people starting and remaining on lifelong ART. With expanded virological monitoring, the number of individuals diagnosed with treatment failure and in need of more costly second‐line regimens will present new challenges. Other indications for switching ART may include toxicity, the potential for significant drug‐drug interactions such as when starting tuberculosis therapy, and pregnancy. Durability, particularly of first‐line regimens, is central to the sustainability of treatment programmes. Therefore, attention should be focused on understanding the factors that influence initial regimen choice, potentially harmful treatment interruptions, and the use of costly second‐ and third‐line regimens.

Patient sex is known to influence antiretroviral treatment decisions. However to date, sex differences in choice of initial regimen, indications for switching, time to switching and choice of second‐line regimens have not been well described. Both males and females, bring unique biological, psychological and social characteristics, which may influence the manifestations of HIV infection and clinical management. Previous analyses have reported that compared to men, women are less likely to have advanced disease and more likely to have a higher CD4 count at ART initiation, and are less likely to be lost to follow‐up 4, 5, 6. Sex differences in choice of initial regimen may be influenced by factors such as local guidelines, drug availability, drug interactions, potential toxicity, pregnancy and pre‐conceptual planning. Similarly, indications for switching may include sex‐specific events such as pregnancy. Early modification of an initial ART regimen has been associated with poor clinical outcomes 7. While the frequency and reasons for change in therapy has been assessed in cohort studies from diverse settings including resource‐rich and ‐limited settings, the differences between men and women have not been comprehensively reported. 8, 9, 10, 11, 12.

Findings from the limited number of studies of sex differences suggest that reasons for ART interruption, choice of regimen and risk of toxicity vary between men and women. In a study from Asia 13, significant differences in choice of initial regimen were reported with more females receiving non‐nucleoside reverse transcriptase inhibitor (NNRTI)‐based combinations. Women were also more likely to change the treatment due to toxicity and side effects. Gender differences in toxicity have been reported for nucleoside analogues 14, NNRTIs 15 and protease inhibitors (PI) 16, though clinical trials have not been able to definitively establish sex differences in toxicity risk. Clinical outcomes such as immunological response, new AIDS events, or death, have not been found to significantly differ by sex in either resource‐rich 17, 18, 19 or ‐limited settings 13.

The primary aim of this study was to describe first‐line ART and CD4 count at ART initiation by sex, time and region. The secondary aim of this study was to analyse time to major change in first‐line ART and time to major interruption in first‐line ART by sex in each region. Particularly in resource‐limited settings, with restricted access to second‐ and third‐line agents, better understanding of time to switching and indications for switching is important for maximizing treatment durability and improving the outcomes.

## 2 Methods

### 2.1 Participants and settings

The International epidemiology Databases to Evaluate AIDS (IeDEA) is a collaborative network of ART programmes with sites in various regions throughout the world. Key clinical data are collected and collated within IeDEA generating large data sets to address high‐priority HIV research questions. Participating cohorts include: Southern, East and West Africa (IeDEA‐Africa), North America (NA‐ACCORD), Caribbean, Central/South America (CCASAnet), and Asia‐Pacific including Australia (IeDEA Asia‐Pacific). All participating cohorts obtained local ethical committee approval to contribute data to the IeDEA collaboration. Countries were characterized as high, middle or low‐income countries based on standard definitions using the gross national income per capita.

### 2.2 Outcomes

The primary outcomes analysed for each region and according to sex were choice of initial antiretroviral regimen, time to switching and time to interruption of the first‐line regimen.

### 2.3 Inclusion criteria and definitions

Patients aged 18 years or older with a known date of ART initiation and a known first‐line regimen who had not previously received ART (with the exception of ART used only for prevention of mother‐to‐child transmission (PMTCT) were included in the study for the period between 2003 and 2014. All data were de‐identified for analysis. A switch in ART was defined as either a change in class of antiretrovirals or a change of at least two agents. An interruption in ART was defined as a documented treatment interruption (i.e. receipt of no antiretrovirals) of at least 30 days. Loss to follow‐up (LTFU) was not considered as a treatment interruption in the main analysis as LTFU could be due to multiple reasons such as transfer out to other ART programmes, death or migration and may not necessarily imply an interruption. However, rates of LTFU (defined as no documented visit for at least twelve months before the closing date of the database) were compared by sex and region.

Patient level variables included sex, age, date of enrolment in cohort, date of last clinic visit, date of death, date of transfer, date of ART initiation, type of first‐line regimen (EFV *vs*. NVP *vs*. PI‐based regimen) and type of nucleoside reverse transcriptase inhibitor (NRTI) backbone (stavudine [d4T] *vs*. zidovudine [AZT] *vs*. TDF *vs*. other NRTI backbone). Pregnancy data were not routinely collected at all sites across the time period of the study or available for inclusion in the final analysis.

### 2.4 Statistical analysis

Descriptive statistics were used to describe first‐line ART and CD4 count at ART initiation by sex, calendar year and region.

To analyse time to switching in first‐line ART and time to interruption in first‐line ART by sex in each region, follow‐up time started at the date of ART initiation and ended at either the first outcome or was censored at the end of available follow‐up. We also assessed whether there was an interaction between region and sex variables. Cox regression models were used to adjust for the following variables: age and CD4 count at ART initiation and mode of exposure (injecting drug use *vs*. other) where available. Mode of exposure was only available for NA‐ACCORD, CCASAnet and IeDEA Asia‐Pacific. Given the heterogeneity between regions, we present the data individually by each region.

Two sensitivity analyses were performed: (i) excluding sites for potential misclassification of treatment interruption (namely two sites from Southern Africa as these sites pre‐populate their ART tables and could potentially record an “interruption” due to recording error) and (ii) excluding women aged <35 years to try and account for switching or interruption due to pregnancy. Change to a tenofovir (TDF)/lamivudine or emtricitabine (XTC) + Efavirenz (EFV) regimen from other NRTI and NNRTI regimens was not counted as a change.

## 3 Results

The combined cohort data set for this study comprised of 715,252 participants across seven regions including both low‐ and high‐income settings. First‐line ART varied according to setting and sex, with changes over time (Table 1). Efavirenz was more likely to be included in the first‐line regimen in men compared with women but the proportion of women initiating efavirenz‐based regimens increased in many regions from 2010 onwards. Protease inhibitors were rarely included in first‐line regimens in low‐income settings compared to high‐income settings for both men and women.

**Table 1:** First‐line antiretroviral therapy according to region and sex over three time periods

Time period	First‐line ART regimen	2003 to 2005		2006 to 2009		2010 to 2014	
Setting		Males N (%)	Females N(%)	Males N (%)	Females N(%)	Males N (%)	Females N(%)
Asia/Pacific Low/low middle‐income settings N = 17,282	EFV	511 (21.6)	134 (15.2)	2047 (48.4)	698 (29.7)	2699 (60.5)	1381 (45.7)
	NVP	1667 (70.7)	642 (73.2)	2024 (47.9)	1427 (60.8)	1613 (36.2)	1463 (48.5)
	PI	42 (1.7)	17 (1.9)	84 (2.0)	42 (1.8)	79 (1.8)	73 (2.4)
	TDF	18 (0.8)	5 (0.6)	244 (5.8)	80 (3.4)	1717 (38.5)	1046 (34.7)
	AZT/3TC	585 (24.8)	171 (19.5)	1559 (36.7)	616 (26.3)	1579 (35.4)	1064 (35.2)
	D4T/3TC	1692 (71.8)	634 (72.3)	2321 (55.0)	1457 (62.1)	1031 (23.1)	736 (24.4)
	Other NRTI backbone	35 (1.5)	61 (6.7)	62 (1.5)	183 (7.8)	77 (1.7)	127 (4.2)
Asia/Pacific Upper‐middle/high‐income settings N = 7394	EFV	312 (35.7)	89 (20.2)	771 (40.8)	148 (24.5)	1366 (67.4)	148 (47.4)
	NVP	268 (30.7)	264 (60.0)	851 (45.0)	385 (63.4)	267 (13.2)	104 (33.3)
	PI	274 (31.2)	73 (16.6)	227 (12.0)	63 (10.4)	337 (16.6)	53 (17.0)
	TDF	67 (7.7)	29 (6.6)	306 (16.2)	85 (14.1)	1020 (50.3)	113 (36.2)
	AZT/3TC	282 (32.3)	74 (16.8)	703 (37.2)	166 (27.4)	411 (20.3)	69 (22.1)
	D4T/3TC	374 (42.8)	290 (65.9)	705 (37.3)	332 (54.9)	174 (8.6)	87 (27.9)
	Other NRTI backbone	150 (17.2)	47 (10.7)	179 (9.5)	21 (3.5)	421 (20.8)	41 (13.1)
Southern Africa N = 478,336	EFV	8576 (39.4)	8524 (28.0)	50,279 (52.9)	60,432 (40.2)	44,772 (68.8)	60,400 (52.7)
	NVP	12,575 (57.8)	21,040 (69.0)	42,609 (44.8)	86,783 (57.7)	18,657 (28.7)	52,234 (45.5)
	PI	630 (2.9)	1000 (3.3)	2269 (2.4)	3259 (2.2)	1542 (2.4)	2038 (1.8)
	TDF	23 (0.1)	58 (0.2)	19,269 (20.3)	24,574 (16.4)	28,524 (43.8)	46,582 (40.6)
	AZT/3TC	7684 (35.3)	7502 (24.6)	14,109 (14.9)	20,732 (13.8)	4623 (7.1)	11,442 (10.0)
	D4T/3TC	13,915 (64.0)	22,779 (74.7)	60,353 (63.5)	102,806 (68.4)	18,328 (28.2)	30,054 (26.2)
	Other NRTI backbone	148 (0.7)	177 (0.6)	1,476 (1.6)	2403 (1.6)	13,660 (21.0)	26,725 (23.3)
East Africa N = 92,054	EFV	883 (21.2)	1105 (13.4)	4349 (25.3)	5338 (16.9)	4440 (44.4)	7516 (41.5)
	NVP	3316 (79.6)	7115 (86.4)	12,893 (75.1)	25,818 (82.0)	5664 (56.6)	10,995 (60.7)
	PI	0 (0)	0 (0)	2 (0.01)	571 (1.8)	0 (0)	11 (0.06)
	TDF	42 (1.0)	102 (1.2)	281 (1.6)	455 (1.4)	3745 (37.4)	6680 (36.9)
	AZT/3TC	404 (9.7)	620 (7.5)	5165 (30.1)	8598 (27.3)	4304 (43.0)	7636 (42.2)
	D4T/3TC	3742 (89.8)	7775 (92.0)	11,780 (68.6)	22,550 (71.6)	1972 (19.7)	3839 (21.2)
	Other NRTI backbone	11 (0.3)	15 (0.2)	29 (0.2)	65 (0.2)	42 (0.4)	68 (0.4)
West Africa N = 52,123	EFV	2097 (46.2)	3285 (42.6)	4292 (45.1)	4623 (25.5)	1740 (45.8)	2305 (30.3)
	NVP	1659 (36.5)	3403 (44.2)	4705 (49.4)	12,444 (68.6)	1762 (46.4)	4705 (61.8)
	PI	637 (14.0)	804 (10.4)	452 (4.7)	850 (4.7)	260 (6.8)	491 (6.5)
	TDF	56 (1.2)	82 (1.1)	1965 (20.6)	2905 (16.0)	1091 (28.7)	1825 (24.0)
	AZT/3TC	1535 (33.8)	2251 (29.2)	3930 (41.2)	7357 (40.6)	1920 (50.5)	4124 (54.2)
	D4T/3TC	2790 (61.4)	5149 (66.8)	3558 (37.4)	7707 (42.5)	659 (17.3)	1479 (19.4)
	Other NRTI backbone	195 (42.3)	268 (3.5)	150 (1.6)	275 (1.5)	140 (3.7)	211 (2.8)
North America, N = 46,023	EFV	1959 (37.5)	543 (30.3)	3733 (45.8)	844 (32.0)	2834 (47.4)	467 (30.7)
	NVP	358 (6.9)	157 (8.8)	271 (3.3)	93 (3.5)	149 (2.5)	44 (2.9)
	PI	2728 (52.3)	982 (54.7)	3989 (48.9)	1629 (61.8)	2492 (41.7)	846 (55.7)
	TDF	2485 (47.6)	730 (40.7)	6401 (78.5)	1787 (67.8)	5320 (88.9)	1216 (80.1)
	AZT/3TC	1,994 (38.2)	834 (46.5)	788 (9.7)	518 (19.7)	199 (3.3)	170 (11.2)
	D4T/3TC	400 (7.7)	145 (8.1)	79 (1.0)	30 (1.1)	16 (0.3)	4 (0.3)
	Other NRTI backbone	454 (8.7)	140 (7.8)	583 (7.2)	206 (7.8)	238 (4.0)	74 (4.9)
Caribbean, Central and South America N = 22,040	EFV	1756 (63.7)	700 (37.9)	3599 (78.6)	1158 (36.9)	3382 (83.8)	1038 (47.9)
	NVP	570 (20.7)	901 (48.8)	343 (7.5)	1569 (50.0)	128 (3.2)	759 (35.0)
	PI	361 (13.1)	135 (7.3)	597 (13.0)	366 (11.7)	489 (12.1)	356 (16.4)
	TDF	62 (2.3)	50 (2.7)	640 (14.0)	218 (7.0)	1642 (40.7)	1003 (46.3)
	AZT/3TC	2261 (82.0)	1556 (84.2)	3321 (72.5)	2441 (77.8)	1941 (48.1)	1010 (46.6)
	D4T/3TC	274 (9.9)	207 (11.2)	381 (8.3)	413 (13.2)	108 (2.7)	67 (3.1)
	Other NRTI backbone	182 (6.6)	70 (3.8)	264 (5.8)	105 (3.6)	351 (8.7)	93 (4.3)

% are column percentages (percentages from the total in the given time‐period and gender).

EFV, Efavirenz; NVP, nevirapine; PI, protease inhibitor; TDF, tenofovir; 3TC, lamivudine; AZT, zidovudine; D4T, stavudine.

More specifically, in the Asia‐Pacific region (including low‐, middle‐ and high‐income countries) nevirapine was the NNRTI more commonly prescribed in the first ART regimen in the earlier time period (2003 to 2005) for both men and women but this declined in the later time period (2010 to 2014) with efavirenz as the most commonly prescribed NNRTI. Not surprisingly, the prescribing of a D4T/3TC backbone declined significantly over time while TDF‐containing regimens increased. A similar pattern in NNRTI prescribing was seen in the Southern Africa and East Africa regions. In contrast, West Africa had a relatively high proportion of clients started on efavirenz across all time periods with little change in proportion of clients on nevirapine over time. And unlike other regions AZT/3TC still remained the preferred NRTI backbone even for the most recent time period (2010 to 2014) for both men and women.

Compared to other regions North America used very little nevirapine as the preferred NNRTI in the initial regimen irrespective of sex (<10% across all time periods) and used very high levels of TDF (>80% in both men and women) in the most recent time period (2010 to 2014). Finally the Caribbean, Central and South America reported consistently higher use of efavirenz compared with nevirapine in men compared with women across all time periods and similar to North America, tenofovir inclusion in the initial regimen increased from <3% in 2003 to 2005 to >40% in 2010 to 2014 in both men and women.

The median CD4 count at ART initiation was, in all cases, lower in men compared to women in each region and time period with the exception of Asia‐Pacific sites and North America from 2010 onwards (Table 2).

**Table 2:** Median CD4 count cells/μL (IQR) at ART initiation by sex and region over time

Time period	2003 to 2005		2006 to 2009		2010 to 2014	
Setting	Males	Females	Males	Females	Males	Females
Asia/Pacific Low/low‐middle income settings	98 (47 to 174)	123 (52 to 203)	125 (55 to 209)	171 (82 to 241)	140 (45 to 272)	226 (101 to 326)
Asia/Pacific Upper to middle/high‐income settings	97 (28 to 229)	113 (32 to 209)	99 (29 to 225)	127 (41 to 211)	206 (64 to 328)	175 (60 to 289)
Southern Africa	83 (19 to 174)	85 (19 to 170)	77 (15 to 180)	101 (18 to 193)	103 (19 to 216)	149 (27 to 257)
East Africa	90 (29 to 165)	117 (47 to 193)	117 (47 to 189)	140 (68 to 206)	157 (65 to 254)	198 (99 to 290)
West Africa	74 (12 to 182)	94 (14 to 208)	98 (20 to 208)	134 (25 to 257)	101 (18 to 236)	150 (25 to 282)
North America	209 (67 to 354)	244 (105 to 400)	261 (113 to 395)	276 (136 to 428)	349 (180 to 512)	324 (165 to 490)
Caribbean, Central and South America	117 (43 to 222)	141 (52 to 234)	152 (55 to 259)	177 (85 to 267)	203 (73 to 322)	235 (117 to 331)

The median age (IQR) at ART initiation was 35.0 years (28.8 to 42.3). For men the median age (IQR) was 37.3 years (31.1 to 44.6) and for women 33.4 years (27.7 to 40.4).

**Figure 1:**
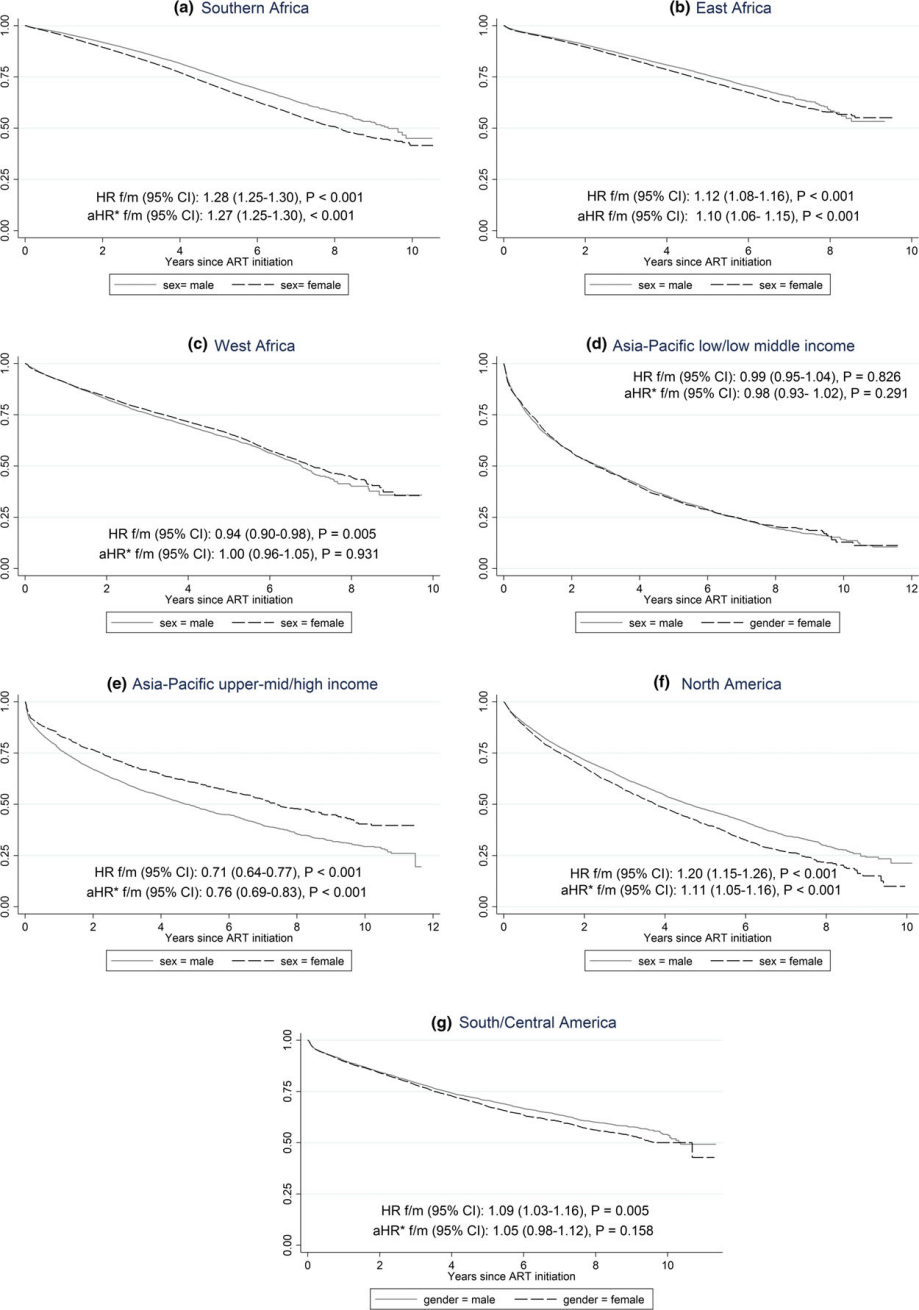
Major change in antiretroviral regimen over time according to region and sex.

Women contributing data from North America and Southern Africa were more likely to have a switch in ART compared to men (Figure 1). In North America, approximately 90% of women had reported a major change in their ART regimen after 10 years compared to closer to 75% of men (*p* < 0.01). In Southern Africa, women were also more likely to have a major change in the ART regimen (*p* < 0.01) but overall after 10 years just under 50% of both men and women were still on their original regimens. Men were more likely to switch ART in Asia/Pacific high‐income countries and East Africa (women were more likely to have a major switch in the first 8 years after treatment initiation, but men are more likely to switch after 8 years, Figure 1). Differences were non‐significant from Caribbean, Central/South America, West Africa and Asia/Pacific low‐income countries.

Overall, 8 years after ART initiation, more than 50% of HIV‐positive males and females from Southern Africa, East Africa, and South and Central America remained on their original regimen without a switch. Males and females from low‐ and middle‐income countries in the Asia/Pacific region were most likely to have had a switch with less than 25% still on their original regimen (Figure 1). This was a similar finding in women from North America with less than 25% on their original regimen after 8 years. These differences occurred irrespective of low‐, middle‐ or high‐income status of the countries contributing data from the region or the most common ART combination prescribed initially in the region.

**Figure 2:**
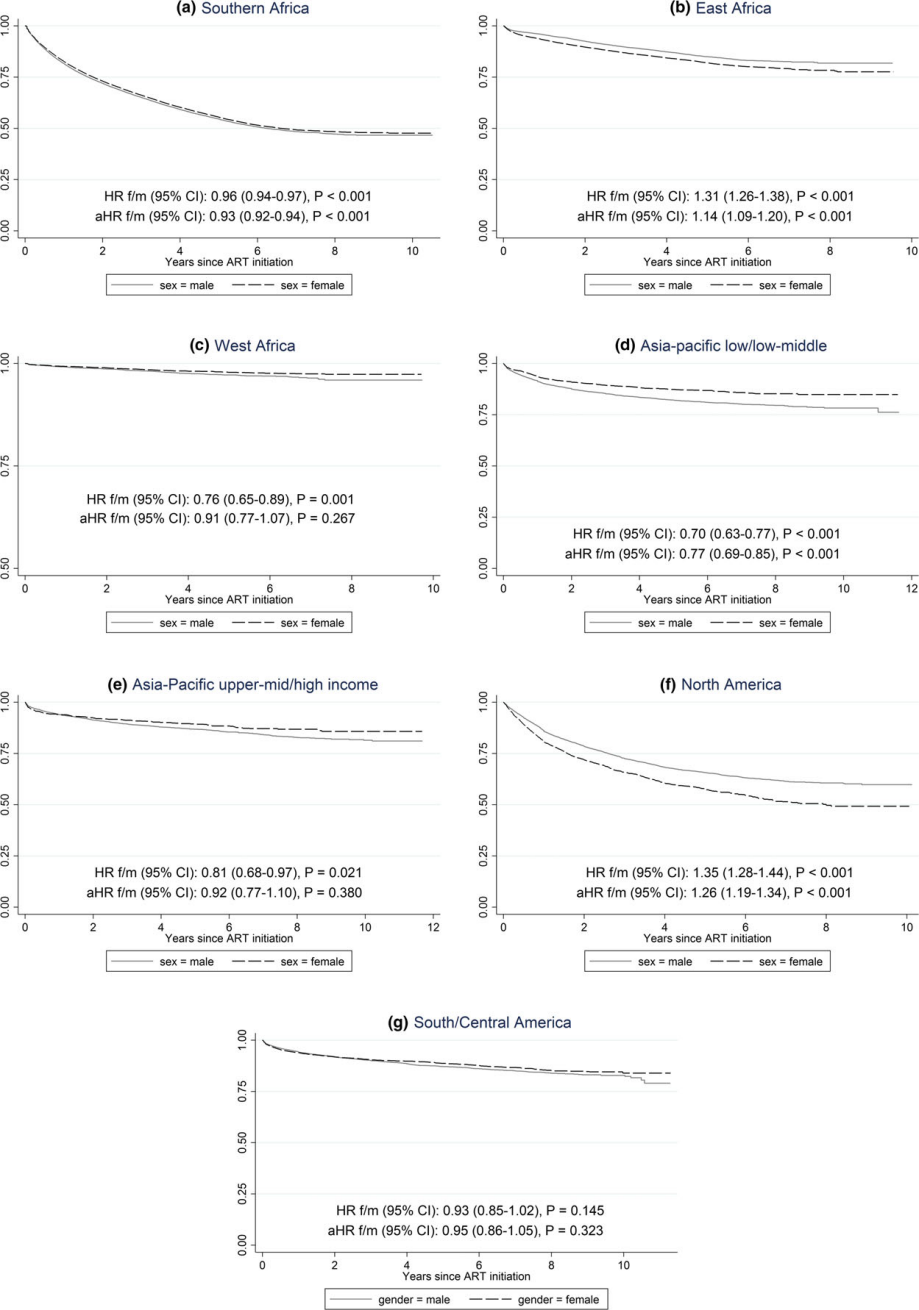
Major interruption in antiretroviral regimen over time according to region and sex.

In terms of treatment interruption there were no significant differences according to sex in South and Central America, West Africa (albeit very low rates of reported treatment interruption) and Asia Pacific (high income). In Asia/Pacific low‐ and lower middle‐income sites men were more likely to have a treatment interruption compared with women (*p* < 0.001) as were men in Southern Africa (*p* < 0.001). Women were more likely to have a treatment interruption compared with males in East Africa and North America (Figure 2). However, in all regions except North America and Southern Africa, greater than 75% of men and women did not report a treatment interruption after 10 years of ART.

## 4 Discussion

Our key findings are that there are regional variations in choice of initial ART regimen according to sex, and that the relative contribution from different antiretroviral classes has changed over time, which likely reflects changes in local, WHO and other guidelines, and availability of individual drugs. Overall, women were more likely than men to switch their ART in North America and Southern Africa and men were more likely to have a treatment interruption in Asia/Pacific high‐income countries and in Southern Africa, although this varied considerably across different regions. Other than in North America and Southern Africa, more than 75% of HIV‐infected individuals had not had a treatment interruption after 10 years irrespective of sex and choice of initial regimen.

We note that efavirenz was more likely to be included in first‐line ART for men compared to women across all regions and all time periods. This likely reflects the historical concerns regarding the potential teratogenicity of efavirenz and the impact of local and international guidelines in place during the period. It was only in 2013 that the WHO guidelines changed to recommend an efavirenz‐based regimens for all, including women of childbearing age and pregnant women. Of note, despite the WHO changes in 2013, all regions with the exception of North America, observed an increase in the proportion of women initiating efavirenz‐containing regimens from 2010 onwards compared to earlier time periods.

Protease inhibitor (PI) based first‐line regimens were infrequent in both men and women in low‐income settings. This is in contrast to North America where PI containing first‐line therapy was used in 40% to 60% of men and women. This occurred across all time periods and likely reflects local guidelines, broader formularies that include PIs, and less prohibitive cost of PIs in a resource‐rich setting.

The median CD4 count at ART initiation by sex was higher in women compared with men across all time periods in low/middle income Asia‐Pacific, East Africa, Southern Africa, West Africa, and the Caribbean, Central and South America. In the remaining regions of Asia‐Pacific high income and North America, the median CD4 count was higher in women compared to men in at least two of the three time periods. Overall this is consistent with other published data 18, 19, 20, 21, 22. This may reflect women presenting earlier (or alternatively men presenting later), women being initiated earlier on treatment or starting treatment earlier because of other sex specific indications such as pregnancy or increased access to health services.

Women were significantly more likely to have a switch in their first‐line regimen compared to men in the Southern African and East Africa cohorts (*p* < 0.001). This difference remained even after restricting the analysis to women >35 years at study entry as a strategy to account for switching due to pregnancy (data not presented). Restricting to women aged greater than 35 years is not an ideal way to account for pregnancy events and could introduce other biases. Unfortunately we did not have individual‐level data on pregnancies and therefore this was one possible way to answer the concern that some treatment decisions are likely influenced by pregnancy. Of note, this was only a sensitivity analysis and not part of the main analysis. Our results were largely similar to the respective figures representing the main analysis (Figures 1 and 2).

Approximately 50% of women in Southern and East Africa cohorts had switched by 8 years of follow‐up; this contrasts with the West Africa regional data, which showed no significant difference according to sex. Similarly, Asia Pacific low/low middle income sites reported no significant difference in rate of switch in ART regimen according to sex. However, it is striking that by 10 years of follow‐up >75% of this cohort (Asia Pacific low/low middle income) had undergone a change in their first‐line regimen. Data from Asia Pacific upper middle/high income sites demonstrated that switching was more likely in males compared to females. This data set has a significant contribution from Australia (17%) which comprises predominantly of men (90% of participant data from Australia to IeDEA Asia‐Pacific is male). Hence, if gender‐specific factors such as pregnancy are driving the earlier switch in ART regimen, then this may explain inconsistent findings in this region compared to the African counterparts. This finding from a high‐income setting in the Asia Pacific region was not a consistent finding in other high‐income settings. In the North American cohort, women were more likely to undergo a switch than men, suggesting socioeconomic status of the region may not be a factor contributing to sex‐specific differences in changes to ART therapy.

Previous studies investigating factors associated with first‐line ART change have reported toxicity as the single most important reason for change 23, 24, 25. The probability of therapy change at one year, has been reported approximating 25% 26, increasing to up to 80% by 6 years. 27. Similarly, reasons for treatment interruption (as opposed to switching) have also been reported in studies to be more often due to toxicity than failure 12. Interestingly, in a study from Latin America and the Caribbean, comprising of 35% females, efavirenz‐based regimens were associated with a lower risk of switching compared to nevirapine‐ and other non‐NNRTI‐based regimens 8. On one hand, this argues against concerns regarding pregnancy and teratogenicity as the reason for switching of first‐line regimens in women, but may actually reflect the recognized higher rates of toxicity from nevirapine‐based regimens in women compared with men. In a study by Monforte et al. 12, sex was an independent factor associated with treatment interruption for toxicity, with men 57% less likely than women to discontinue ART because of toxicity. The findings in our study are similar in terms of proportions of patients who undergo a treatment switch, although unfortunately this study did not explore the reasons for switching. Teasing out the reasons for switch (toxicity, pregnancy, drug interactions or resistance) warrants further investigation and is essential to our understanding of the possible differences between change and treatment interruption as observed in our study according to sex.

Although routine HIV viral load testing may detect early emerging drug resistance and lead to switching of ART regimen, the availability and frequency of this test across different regions of the world remains variable. A Cochrane review 28 evaluated the optimal monitoring strategy for guiding when to switch ART for first‐line treatment failure. Their conclusion was that there was a substantial benefit in terms of mortality and AIDS defining illness if immunological and clinical or virological monitoring was used versus clinical monitoring alone 28. Haas et al. recently examined the rate of switching from first‐line ART across 16 countries in sub‐Saharan Africa. Only 3% of those followed up had switched to second‐line ART; change of ART was not only more common but also occurred earlier with the use of targeted routine viral load testing 11. It is unlikely that the differences reported in our study between men and women in terms of switching is due to the availability (or not) of viral load testing per region. It is expected that viral load testing if available would be accessible equally for men and women in a particular region and therefore switching in relation to availability of testing should be equally distributed according to sex.

In general, the likelihood of a treatment interruption varied according to sex and region. Treatment interruption was more likely for males in Southern Africa, Asia‐Pacific low‐ and middle‐income sites. In West Africa, there was no significant difference according to sex but the data suggest that 90% of participants had no treatment interruption after 10 years of follow‐up which is significantly higher than the other African cohorts. The other regions where there were no significant differences in the rate of treatment interruption according to sex when adjusted for age and CD4 count at ART initiation, first‐line NRTI backbone (AZT *vs* TDF *vs* D4T), type of anchor agent (NVP *vs* EFV *vs* PI) and mode of exposure included high‐income Asia Pacific sites and Caribbean Central/South America. Only in North America were women more likely to have a treatment interruption. An important consideration in interpreting this finding is to ask if it is due to data capture, or due to gender‐specific issues and how these may vary across the different regions sufficiently to explain the inconsistencies.

A major strength of this study is that it analysed a large cohort consortia from a diverse range of settings and regions, but a significant limitation of this paper is the inability to account for important factors unique to women such as pregnancy which are likely to impact on switching and treatment interruption. Women already initiated on ART may change their treatment when they find out they are pregnant. Similarly, women may interrupt their therapy post partum either due to patient or programmatic factors (Option B *vs*. B+). In 2015, the WHO recommended lifelong ART for all pregnant and breastfeeding women regardless of CD4 count. Much of the data in our study included women prior to 2015.

In summary, we have demonstrated that there are sex and regional variations in choice of initial ART, switching and treatment interruption. Given the massive scale up of HIV treatment that has occurred over the last decade, and the anticipated expansion in ART services in the next decade, it is essential to understand the drivers behind these differences. Drivers of treatment change such as pregnancy, toxicity, drug interactions and resistance need to be considered in more detail when exploring reasons for ART change or interruption, and in particular need to be compared between men and women.

## Competing interests

There are no potential conflicts of interest to declare for any of the authors.

## Authors’ Contributions

MLG conceived the project and contributed to manuscript writing. AA conducted the analysis, led interpretations and contributed to manuscript writing. AA was supervised by MGL who also contributed to the analysis and interpretation of results. AGA, ADH, MJG, LMP, ML, CM, MC, PB, NdR, RB, KW‐K contributed data and reviewed the manuscript. All authors have read and approved the final manuscript.

## Funding

This study was supported by the U.S. National Institutes of Health’s National Institute of Allergy and Infectious Diseases, the Eunice Kennedy Shriver National Institute of Child Health and Human Development, and the National Cancer Institute under the following award numbers by region ‐ Asia‐Pacific: U01AI069907; Caribbean, Central and South America (CCASAnet): U01AI069923; Southern Africa: U01AI069924; East Africa: U01AI069911; West Africa: U01AI069919; and North America (NA‐ACCORD): U01AI069918, F31DA037788, G12MD007583, K01AI093197, K23EY013707, K24DA000432, K24AI065298, KL2TR000421, M01RR000052, N02CP055504, P30AI027757, P30AI027763, P30AI027767, P30AI036219, P30AI050410, P30AI094189, P30AI110527, P30MH62246, R01AA016893, R01CA165937, R01DA004334, R01DA011602, R01DA012568, R24AI067039, U01AA013566, U01AA020790, U01AI031834, U01AI034989, U01AI034993, U01AI034994, U01AI035004, U01AI035039, U01AI035040, U01AI035041, U01AI035042, U01AI037613, U01AI037984, U01AI038855, U01AI038858, U01AI042590, U01AI068634, U01AI068636, U01AI069432, U01AI069434, U01AI103390, U01AI103397, U01AI103401, U01AI103408, U01DA036935, U01HD032632, U10EY008057, U10EY008052, U10EY008067, U24AA020794,U54MD007587, UL1RR024131, UL1TR000004, UL1TR000083, UL1TR000454, UM1AI035043, Z01CP010214 and Z01CP010176; contracts CDC‐200‐2006‐18797 and CDC‐200‐2015‐63931 from the Centers for Disease Control and Prevention, USA; contract 90047713 from the Agency for Healthcare Research and Quality, USA; contract 90051652 from the Health Resources and Services Administration, USA; grants CBR‐86906, CBR‐94036, HCP‐97105 and TGF‐96118 from the Canadian Institutes of Health Research, Canada; Ontario Ministry of Health and Long Term Care; and the Government of Alberta, Canada. Additional support was provided to NA‐ACCORD by the Intramural Research Program of the National Cancer Institute. The Kirby Institute is funded by the Australian Government Department of Health and Ageing, and is affiliated with the Faculty of Medicine, UNSW Australia (The University of New South Wales). The funders had no role in study design, data collection and analysis, decision to publish, or preparation of the manuscript. The content is solely the responsibility of the authors and does not necessarily represent the official views of any of the governments or institutions mentioned above. We thank all patients and their families, and all staff at participating sites for preparation of data contributed to this collaborative work. We also thank the IeDEA‐WHO Collaboration for their guidance and expertise.
